# Successful Intensive Management and Serial Clinical Progression of Severe Non-Exertional Heatstroke in a Cat Following Clothes Dryer Entrapment

**DOI:** 10.3390/vetsci13090939

**Published:** 2026-09-10

**Authors:** Ji-Young Lee, Jung-Hyun Song, Kun-Ho Song

**Affiliations:** Department of Veterinary Internal Medicine, College of Veterinary Medicine, Chungnam National University, Daejeon34134, Republic of Korea; 202560386@o.cnu.ac.kr (J.-Y.L.); jh.song@cnu.ac.kr (J.-H.S.)

**Keywords:** heatstroke, cat, non-exertional heatstroke, clothes dryer, fSAA, SIRS, time to treatment, rapid cooling

## Abstract

Heatstroke is a life-threatening veterinary emergency that is rarely reported in cats, particularly when secondary to household hazards such as clothes dryer entrapment. This report details the successful intensive management and clinical progression of a single feline patient presenting with severe non-exertional heatstroke and concurrent blunt force trauma following approximately 20 min of entrapment in an operating dryer. Upon presentation, the cat exhibited severe hyperthermia (43.0 °C), circulatory shock, oral hemorrhage, and generalized seizures. The rapid initiation of active cooling and aggressive hemodynamic resuscitation prevented further life-threatening complications beyond the initial day of seizures, leading to clinical stabilization and discharge on day 6 at the owner’s request, with a full recovery confirmed at a recheck appointment on day 11. Furthermore, serial biomarker tracking during hospitalization revealed a continuous elevation in feline serum amyloid A (fSAA) accompanied by intermittent hyperthermia. This sustained inflammatory response suggests that feline heatstroke shares a pathophysiological mechanism with systemic inflammatory response syndrome (SIRS). Ultimately, this case highlights the hidden but severe dangers of household appliances and suggests that early recognition and the immediate initiation of targeted therapy are critical for achieving favorable outcomes in feline non-exertional heatstroke.

## Simple Summary

This report highlights the dangers of accidental clothes dryer entrapment, a rare but life-threatening cause of non-exertional heatstroke in pet cats. The report describes a single case where a cat was mistakenly trapped inside a running dryer for approximately 20 min to demonstrate the severe consequences of combined thermal and physical injuries. The patient received immediate veterinary care for severe overheating, shock, and seizures, leading to clinical stabilization and discharge at the owner’s request 6 days post-admission, and ultimately, a full recovery confirmed at a recheck appointment on day 11. Additionally, serial monitoring of blood markers during hospitalization revealed a sustained systemic inflammatory response, providing clinical insight into the body’s reaction to severe heat stress. This successful outcome suggests that rapid recognition, immediate cooling, and urgent veterinary care are critical to saving a pet’s life in overheating emergencies. By raising awareness among pet owners about this hidden household hazard and emphasizing the critical need for immediate medical intervention, this study helps families protect their pets from preventable tragedy.

## 1. Introduction

Heatstroke is a life-threatening medical emergency characterized by central nervous system dysfunction and a rapid progression to circulatory failure [1,2,3]. While exertional heatstroke is typically associated with strenuous physical activity, non-exertional (environmental) heatstroke occurs following prolonged exposure to high ambient temperatures in poorly ventilated, confined spaces [4,5,6]. Common domestic examples of the latter include confinement in closed vehicles [6] or, less frequently reported but equally perilous, accidental entrapment within operating clothes dryers [4]. Clothes dryer entrapment is a particularly unique and severe environmental hazard because it exposes the animal not only to extreme thermal stress but also to mechanical blunt force trauma from the rotating drum [4].

In the pathophysiology of heatstroke, overwhelmed compensatory thermoregulatory mechanisms trigger a cascade of widespread endothelial injury and SIRS, which can subsequently progress to multiorgan dysfunction syndrome (MODS), leading to high morbidity and mortality rates [1,2,3,7,8,9]. In critically ill feline patients, acute-phase proteins—particularly fSAA—have been demonstrated to significantly increase in response to SIRS [10]. Therefore, serial tracking of acute-phase proteins, such as fSAA, may provide valuable insights into the ongoing systemic inflammatory mechanisms triggered by severe thermal insult [8]. Although the pathophysiology and clinical management of heatstroke have been extensively documented in humans and dogs [3,6,11,12,13], specific data regarding feline heatstroke remain exceedingly scarce, primarily limited to small case series such as those reported by Cudney et al. (2021) [4]. Consequently, establishing definitive prognostic factors or applying temporal frameworks directly from human medicine to felines requires caution [4].

This report aims to address this knowledge gap by detailing the successful intensive management of a feline patient following an estimated 20-minute entrapment in an operating clothes dryer. By describing the clinical progression, the management of combined thermal and mechanical injuries, and the serial tracking of fSAA, this case highlights the severe risks posed by household hazards. Furthermore, it suggests that despite the lack of established species-specific prognostic models, the rapid initiation of intensive therapy may mitigate life-threatening complications and yield successful clinical outcomes in severe feline non-exertional heatstroke.

## 2. Case Description

**Figure 1:**
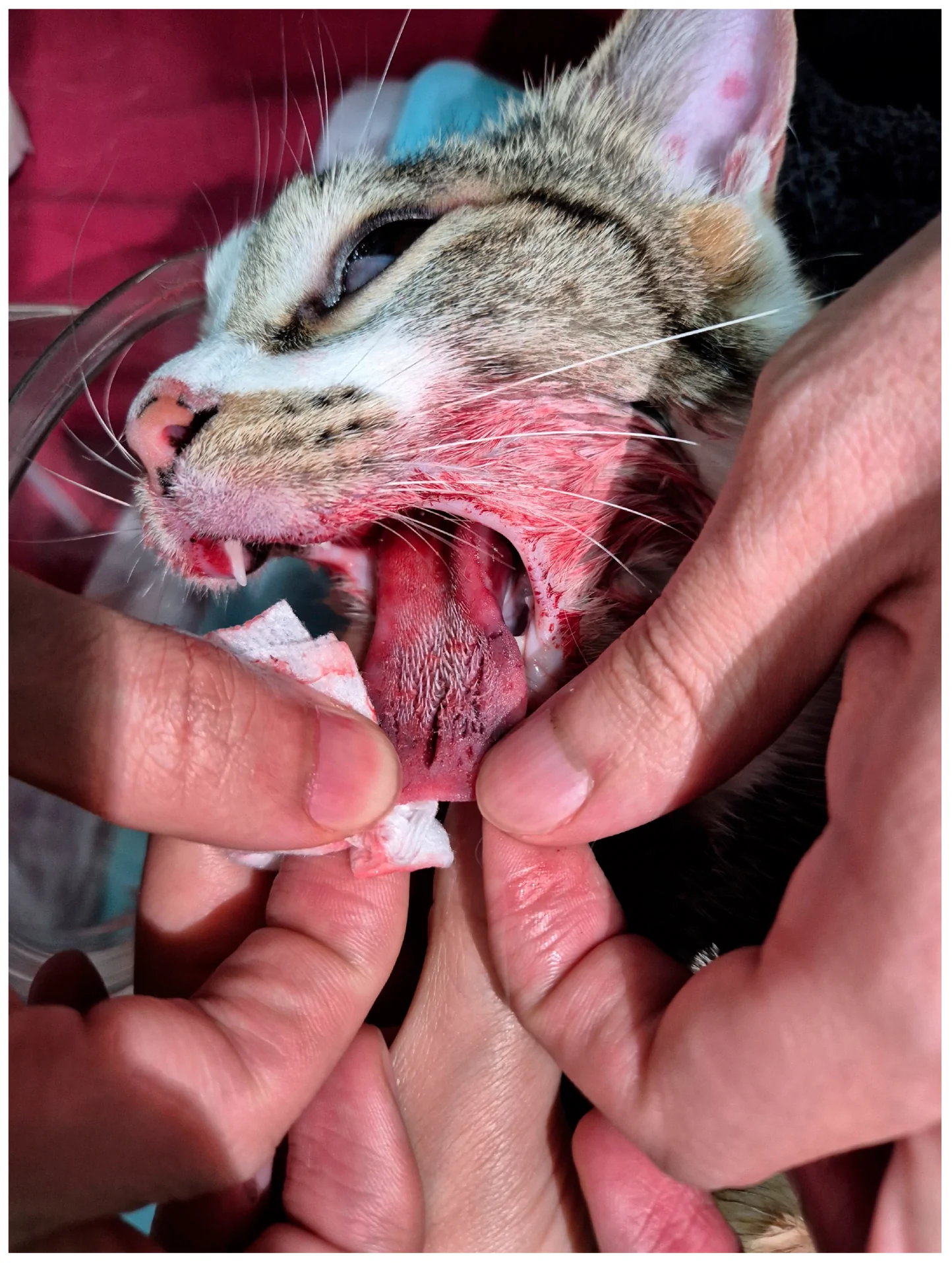
Gross presentation of severe oral hemorrhage in a feline patient following entrapment in a clothes dryer. Note the diffuse perioral and mucosal bleeding, accompanied by a prominent, deep longitudinal laceration on the dorsal aspect of the tongue resulting from mechanical trauma.

A 3-year-old spayed female Korean Domestic Shorthair cat weighing 5.4 kg presented to the emergency department after being discovered mid-cycle following an estimated 20-minute entrapment in an operating household clothes dryer. Although the exact model and specific program settings of the appliance were unavailable, the internal temperature was presumed to be within the standard residential drying range of 50 to 60 °C. The owner immediately extracted the cat and transported her to the hospital by car, with an estimated delay of 10 min from extraction to arrival. At presentation, physical examination revealed depressed mentation and severe hyperthermia. These conditions were evidenced by a rectal temperature of 43.0 °C (which represented the maximum display limit of the digital thermometer used), marked tachypnea (120 breaths/min), and severe hypotension, with a systolic blood pressure (SBP) of 30 mmHg measured via Doppler ultrasonography (Vet-Dop2, Vmed Tech., Seattle, WA, USA) using a size 2 cuff placed on the right forelimb. Assessment of hydration status revealed mildly delayed skin turgor and a capillary refill time of 1 to 2 s, although mucous membrane evaluation was precluded by severe oral hemorrhage. The hemorrhage, which was suspected to be secondary to a lingual laceration, was immediately noted and cleared via mechanical suctioning (Figure 1).

Within 3 min of admission, active cooling protocols, consisting of evaporative cooling with room-temperature tap water and fans, were initiated rapidly. To facilitate safe cooling and control severe agitation, sedation was administered using propofol (10 mg total dose, IV; Freefol-MCT Inj., Daewon Pharm. Co., Seoul, Republic of Korea). Simultaneously, aggressive hemodynamic resuscitation was performed using Lactated Ringer’s solution (10 mL/kg, IV over 20 min; inno.N Hartmann’s Sol., HK inno.N Co., Cheongju, Republic of Korea). Following the initial fluid bolus, the patient’s blood pressure was reassessed; as severe hypotension (SBP of 50 mmHg) persisted, a continuous rate infusion (CRI) of dobutamine (5 μg/kg/min, IV; Myungmoon Dobutamine Inj., Myungmoon Pharm. Co., Seoul, Republic of Korea) was subsequently initiated to improve myocardial contractility and thereby promote an increase in blood pressure.

Core body temperature was frequently monitored via intermittent rectal thermometer readings every 10 to 20 min. Following 20 min of active cooling, the patient’s rectal temperature precipitously dropped from 43.0 °C to 35.0 °C (approximately 24 °C/h). Due to this rapid decrease, active cooling was immediately discontinued, and gentle warming with blankets was initiated to prevent further hypothermia and restore thermal homeostasis. Subsequent core temperatures were maintained between 38.0 °C and 39.0 °C. One hour after admission, hemodynamic stabilization was achieved as the SBP increased to 120 mmHg, allowing for the discontinuation of the dobutamine CRI.

Approximately two hours post-admission, following emergence from the initial propofol sedation, the patient developed generalized tonic–clonic seizures accompanied by involuntary urination and paddling. The patient experienced a total of four discrete episodes of generalized seizures, which were successfully arrested with repeated administrations of midazolam (0.2 mg/kg, IV, PRN; total of four doses; Midacum Inj., Myungmoon Pharm. Co., Seoul, Republic of Korea). Despite the cessation of generalized seizures, partial seizures and persistent nystagmus were subsequently observed. Due to these continuous and refractory neurological signs, levetiracetam (30 mg/kg, IV, q8h; Keppra Sol., UCB Pharm. Co., Brussels, Belgium) was added to the therapeutic protocol, and medical management for suspected intracranial hypertension was initiated using 7.5% hypertonic saline (4 mL/kg, IV, slow infusion over 15 min), alongside dexamethasone (0.2 mg/kg, IV; Jeil Dexamethasone Inj., Jeil Pharm. Co., Seoul, Republic of Korea) to mitigate severe systemic inflammation and neuroinflammation. No further seizure activity was observed following these interventions.

**Figure 2:**
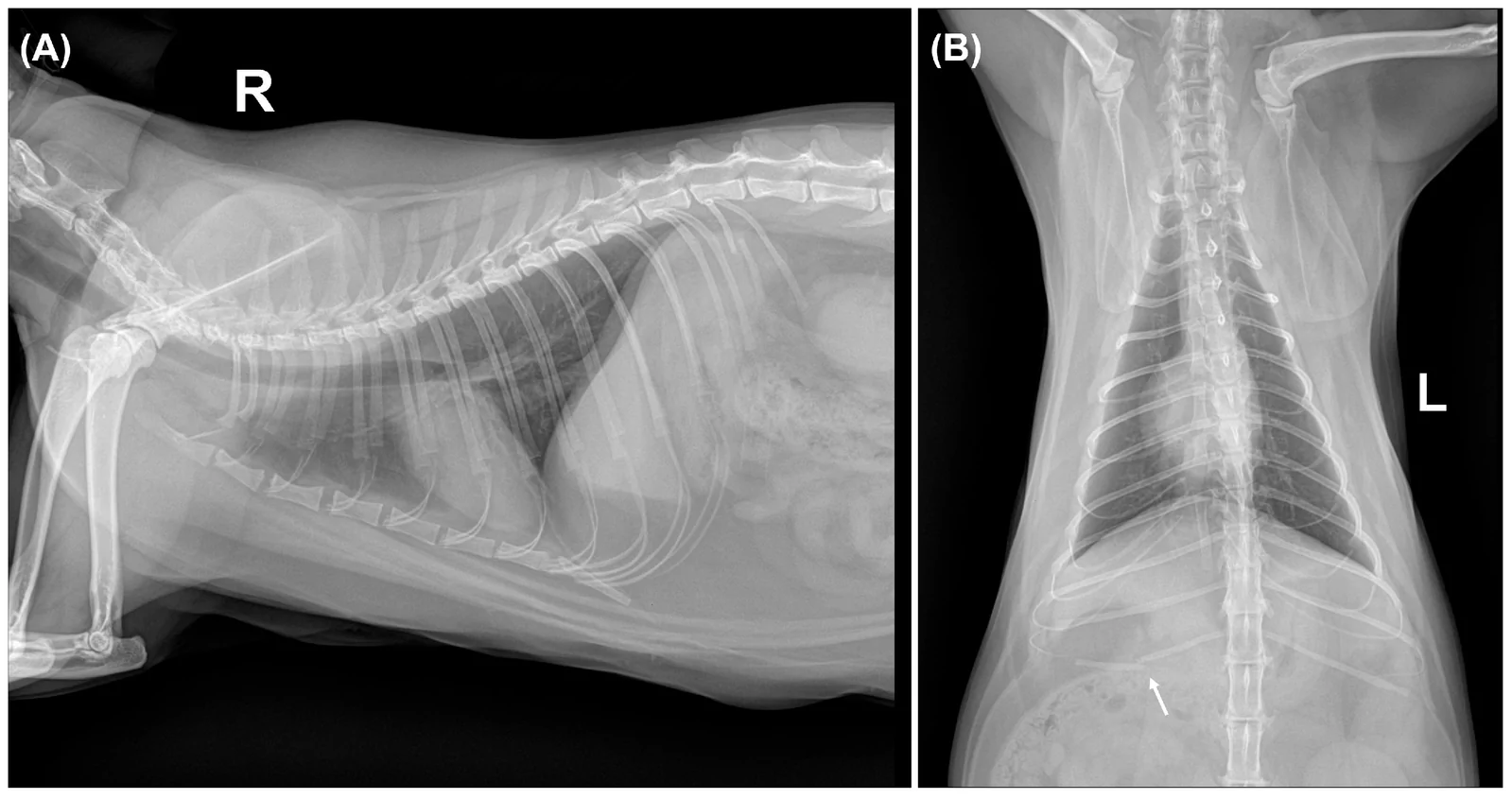
Thoracic radiographs of the feline patient, taken immediately post-stabilization following clothes dryer entrapment. (**A**) Lateral view demonstrating a generalized mild interstitial to alveolar pattern consistent with early pulmonary edema. (**B**) Ventrodorsal view demonstrating a non-displaced fracture of the right 13th rib (arrow). R, right; L, left.

Concurrent with stabilization, thoracic radiographs were performed to assess for heat-induced pulmonary injury and traumatic lesions sustained during entrapment. Radiographs (ECO-CM-N, EcoRay Co., Seoul, Republic of Korea) revealed generalized mild pulmonary edema and confirmed a non-displaced fracture of the right 13th rib (Figure 2).

To provide a comprehensive overview of the clinical progression, the serial laboratory findings—including complete blood count, serum biochemistry, renal parameters, and coagulation profiles—are summarized in Table 1. Initial blood work, including CBC (ProCyte Dx Hematology Analyzer, IDEXX Laboratories, Westbrook, ME, USA), serum biochemistry (Catalyst Dx Chemistry Analyzer, IDEXX Laboratories, Westbrook, ME, USA), electrolyte analysis (ALB80 FLEX BASIC, Central Medical Co., Seoul, Republic of Korea), and a coagulation panel (qLabs Vet Coag Panel2, Micropoint Biotechnologies Inc., Seoul, Republic of Korea), revealed a hematocrit (HCT) of 63.1%, marked elevations in hepatocellular leakage enzymes (alanine aminotransferase [ALT], 3188 U/L; aspartate aminotransferase [AST], 4018 U/L), and unmeasurably high creatine kinase (CK), while other parameters including coagulation profiles, renal values, and total bilirubin were within normal limits.

Following initial stabilization and diagnostic evaluation, a urinary catheter was placed to monitor urine output, alongside continuous temperature monitoring and regulation. For ongoing supportive care, the patient was placed in an oxygen cage (fraction of inspired oxygen [FiO_2_] of 40%), and intravenous fluid therapy was maintained with Lactated Ringer’s solution (48 mL/kg/d). The pharmacological regimen included cefazolin (22 mg/kg, IV, q12h; Cefazolin Inj., Chong Kun Dang Pharm. Co., Seoul, Republic of Korea), metronidazole (10 mg/kg, IV, q12h; Metrinal Inj., Daihan Pharm. Co., Seoul, Republic of Korea), N-acetylcysteine (70 mg/kg, IV, slow infusion over 20 min, q8h; Muteran Inj., Hanwha Pharm. Co., Seoul, Republic of Korea), levetiracetam (30 mg/kg, IV, q8h; Keppra Sol., UCB Pharm. Co., Brussels, Belgium), 7.5% hypertonic saline (4 mL/kg, IV, slow infusion over 15 min, q12h), pantoprazole (1 mg/kg, IV, q12h; Pantoline Inj., Dong-A ST Co., Seoul, Republic of Korea), and maropitant (1 mg/kg, IV, q24h; Cerenia, Zoetis, Parsippany, NJ, USA), alongside eye lubrication (q6h; Liposic, Baush Health, Quebec, QC, Canada).

On day 2 of hospitalization, aside from intermittent hyperthermia, the patient’s vital signs remained stable. The patient exhibited signs of pain, prompting the addition of butorphanol (0.2 mg/kg, IV, q8h; Butophan Inj., Myungmoon Pharm. Co., Seoul, Republic of Korea) to the treatment regimen. Voluntary enteral nutrition and defecation were absent at this time. Urine output via the indwelling catheter ranged from 1.0 to 2.5 mL/kg/h. Repeat bloodwork showed a precipitous drop in HCT to 33.7%, mild elevation in fSAA to 5.3 μg/mL, and an unmeasurably high creatine kinase (CK). Hepatic enzymes showed a decreasing trend, with ALT at 1778 U/L and AST at 1997 U/L.

By day 3, the cat initiated voluntary intake of a gastrointestinal dry diet, and urine output was maintained at 1.8 to 3.4 mL/kg/hr via the indwelling catheter, but intermittent hyperthermia persisted. The lingual laceration was managed conservatively; no further oral hemorrhage occurred, and the lesion healed uneventfully without the need for surgical intervention. Serial bloodwork revealed a further decline in HCT to 27.2%, mild thrombocytopenia (117,000/μL), and a progressive increase in fSAA to 16.1 μg/mL. Hepatic enzymes continued to decrease (ALT 1136 U/L, AST 1887 U/L), but total bilirubin transiently increased (1.1 mg/dL). CK remained unmeasurable. Given the absence of further neurologic signs or indicators of intracranial hypertension, the hypertonic saline was discontinued after a total of five doses. Enrofloxacin (5 mg/kg, IM, q24h; Baytril, Elanco, Indianapolis, IN, USA) and oral hepatoprotectants, including silymarin (10 mg/kg, PO, q12h; Legalon, Bukwang Pharm. Co., Seoul, Republic of Korea), ursodeoxycholic acid (10 mg/kg, PO, q12h; Ursa, Daewoong Pharm. Co., Seoul, Republic of Korea), and S-adenosylmethionine (20 mg/kg, PO, q24h; Sadenin, Chodang Pharm. Co., Seoul, Republic of Korea), were added to the therapeutic protocol to support ongoing hepatic recovery.

On day 4, the urinary catheter was removed, and the patient subsequently exhibited normal voluntary urination. The patient’s voluntary appetite continued to improve, and the hyperthermia was gradually decreasing, although the core temperature remained mildly elevated alongside otherwise stable vital parameters. Bloodwork on day 4 showed a stable HCT, a continued decline in hepatic enzymes alongside a normalization of total bilirubin (0.3 mg/dL), a measurable CK of 8614 U/L, and a mild increase in fSAA to 18.8 μg/mL. By day 5, the cat exhibited a normal appetite, and core body temperature normalized. Laboratory findings demonstrated a stable HCT, further decreases in hepatic enzymes and CK (4154 U/L), and an initial decline in fSAA (17.3 μg/mL). Due to consistent clinical improvement, the patient was discharged on day 6 at the owner’s request. At a recheck appointment 5 days post-discharge (day 11), bloodwork revealed complete resolution of the anemia, as well as normalization of hepatic enzymes and inflammatory markers. All medical treatments were subsequently concluded.

## 3. Discussion

Heatstroke is a life-threatening medical emergency characterized by a rapid progression from an initial compensated thermoregulatory state to decompensated circulatory failure [1,2]. This critical transition triggers a severe pathophysiological cascade involving tissue hypoxia, gastrointestinal barrier disruption, and systemic inflammatory and coagulatory activation, which ultimately leads to MODS [2,3,5,7]. Because the onset of these irreversible cellular injuries occurs rapidly, immediate clinical recognition and aggressive intervention are generally recommended to halt the progression of systemic decompensation [1,2,12,14]. In critically ill feline patients, acute-phase proteins—particularly fSAA—have been demonstrated to significantly increase in response to SIRS [10]. Because the profound inflammatory cascade triggered by severe heatstroke shares underlying mechanisms with SIRS, serial tracking of fSAA may provide valuable insights into the ongoing systemic inflammatory response induced by severe thermal insult [8].

Non-exertional heatstroke in felines, particularly secondary to clothes dryer entrapment, remains exceedingly rare [4]. Importantly, clothes dryer entrapment differs from typical environmental heat exposure (e.g., confined vehicles [6]) because it inflicts a unique, combined injury pattern involving both extreme thermal stress and mechanical blunt force trauma. In this patient, the tumbling mechanism within the rotating drum likely caused the traumatic lingual laceration and rib fracture. This dual insult—thermal and physical—is highly consistent with the findings of Cudney et al. (2021) [4], who also reported concurrent thermal and blunt force injuries in feline clothes dryer entrapment cases. Furthermore, the tissue injury resulting from blunt force trauma can exacerbate the release of pro-inflammatory cytokines, compounding the SIRS induced by extreme hyperthermia [2,3,10]. Consequently, this case suggests that the successful management of feline clothes dryer entrapment requires a comprehensive approach that simultaneously addresses both the mechanical trauma and the severe systemic effects of heatstroke [15].

Regarding the rapid temperature decline observed during initial stabilization, reaching 35.0 °C was not an intended therapeutic endpoint, but rather an unintentional overshoot (iatrogenic hypothermia) resulting from an extreme cooling rate. This precipitous drop can be attributed to a combination of severe pathophysiological derangements and feline-specific physical characteristics. First, severe heatstroke is known to induce direct thermal injury to the hypothalamus, effectively paralyzing the central thermoregulatory set-point; this renders the patient highly susceptible to uncontrolled temperature shifts once external cooling is initiated [16]. Second, because cats have a significantly higher surface area-to-volume ratio than humans or larger dogs, they are highly vulnerable to rapid heat loss when exposed to moisture and subsequent external evaporative cooling [17]. Lastly, the patient presented in a state of decompensated shock characterized by profound peripheral vasoconstriction and compromised systemic perfusion. In such states of impaired microvascular distribution, core heat cannot be efficiently transferred to the periphery, making the physiological response to external cooling erratic and difficult to titrate [15,18]. In this context, the immediate cessation of active cooling and the prompt initiation of passive warming were critical to successfully stabilizing this patient’s core temperature. Therefore, to prevent iatrogenic hypothermia in feline heatstroke patients presenting with shock and compromised perfusion, clinicians must employ frequent core temperature monitoring and carefully titrated cooling protocols [13,15].

To provide optimal hemodynamic support, a continuous rate infusion of dobutamine was initiated after a reassessment of the patient’s volume status. Following the initial 10 mL/kg crystalloid bolus, the patient’s perfusion parameters (e.g., capillary refill time, mucous membrane color) showed partial improvement, suggesting an incomplete resolution of hypovolemia; however, severe hypotension and hypothermia persisted. The patient presented with cold extremities, indicative of profound peripheral vasoconstriction. Consequently, peripheral vasopressors were withheld to avoid exacerbating peripheral tissue hypoperfusion [19]. Furthermore, the markedly elevated CK levels indicated severe skeletal muscle injury, reflecting the profound systemic impact of the thermal insult [14]. Given this severity, concurrent heat-induced myocardial impairment was also considered, as severe overheating is known to cause direct thermal damage to cardiomyocytes, drastically reducing myocardial contractility [20]. Therefore, rather than inducing further vasoconstriction, dobutamine—a positive inotrope—was selected as the most appropriate therapeutic agent to directly enhance myocardial contractility and successfully restore systemic blood pressure [15,21,22].

Compared to the case series by Cudney et al. (2021) [4], where cats required intensive management for multiorgan complications such as coagulopathy and acute kidney injury, these specific sequelae were not observed in our patient. Instead, the clinical challenges in our case were primarily driven by severe mechanical trauma, presenting as a deep lingual laceration, and profound neurological dysfunction. Consequently, while the previous report highlighted general supportive care for mucosal ulcerations and neurological signs, our patient necessitated a highly targeted pharmacological approach to manage decompensated circulatory shock and refractory seizures. Specifically, we utilized a dobutamine continuous rate infusion to restore hemodynamics without worsening peripheral vasoconstriction. Furthermore, to address the profound neurological complications, we administered hypertonic saline for suspected intracranial hypertension, levetiracetam to arrest refractory seizures, and dexamethasone to mitigate severe neuroinflammation and systemic inflammatory responses. This comparison underscores that clothes dryer entrapment can present with highly variable clinical trajectories; therefore, even in the absence of typical multiorgan failure, successful recovery hinges on a rapid, multimodal approach tailored to the patient’s specific hemodynamic and neurological crises.

The dramatic drop in hematocrit from 63.1% at admission to 27.2% by day 3 requires careful clinical interpretation. Rather than resulting from a single catastrophic hemorrhagic event, this progressive anemia was a multifactorial process driven heavily by fluid dynamics. Initially, severe dehydration and fluid shifts caused profound hemoconcentration (63.1%), effectively masking the patient’s true red blood cell mass. As aggressive intravenous fluid resuscitation restored intravascular volume, the resulting hemodilution unmasked an underlying anemic state. This decline in hematocrit was further compounded by direct blood loss from the vascular lingual laceration and iatrogenic blood loss from serial phlebotomy [8,9]. Furthermore, the profound hyperthermia likely inflicted direct thermal injury to the erythrocytes. Temperatures exceeding 40 °C can denature essential cell membrane proteins, compromising the structural integrity and biconcave shape of the red blood cells. This thermal degradation leads to a loss of cellular deformability and increased fragility, often resulting in abnormal morphologies. Consequently, these stiffened, fragile erythrocytes are highly susceptible to premature clearance through low-grade intravascular hemolysis driven by mechanical shear stress and rapid extravascular phagocytosis by the mononuclear phagocyte system [15]. In our case, this heat-induced hemolytic process is strongly supported by the transient elevation of total bilirubin to 1.1 mg/dL on day 3. Its rapid normalization to 0.3 mg/dL by day 4 reflects a single wave of damaged erythrocyte clearance by a recovering hepatobiliary system, rather than progressive hepatic failure. Importantly, severe heatstroke frequently triggers consumptive coagulopathies, such as disseminated intravascular coagulation (DIC), or acute kidney injury (AKI)—both of which can drastically exacerbate anemia through hemolysis or internal hemorrhage [8,9]. However, this patient maintained stable serial coagulation and renal profiles throughout hospitalization. The absence of overt DIC or AKI indicates that the anemia was primarily of hemodynamic, mechanical, and thermal origin, demonstrating that the patient did not progress to the uncompensated, multiorgan-failure stages typically associated with severe thermal injury.

The empirical use of prophylactic antibiotics and targeted neurological therapies in this patient warrants specific discussion. Regarding the antimicrobial approach, although no active systemic infection was documented at admission, broad-spectrum antimicrobial therapy was initiated for two primary reasons. First, the patient sustained a severe lingual laceration, presenting a high risk for secondary bacterial infection from the normal oral flora. Second, and more importantly, the pathophysiology of severe heatstroke involves profound gastrointestinal ischemia and mucosal barrier disruption. This breakdown facilitates the translocation of enteric bacteria and endotoxins into the systemic circulation, significantly increasing the risk of SIRS and secondary sepsis [7]. Therefore, preemptive antimicrobial therapy was deemed a critical component of the early management strategy.

Furthermore, the administration of 7.5% hypertonic saline was instituted to manage suspected intracranial hypertension and cerebral edema [23,24]. Concurrently, dexamethasone was administered to mitigate the profound systemic inflammatory response and blood–brain barrier (BBB) disruption caused by severe heatstroke [8]. In heatstroke, profound hyperthermia and subsequent endothelial injury can severely compromise the BBB, leading to rapid cerebral edema—a major contributor to heatstroke-related mortality [9,14,23,24]. To counter this, clinical guidelines and current evidence suggest that the administration of glucocorticoids, such as dexamethasone, can effectively suppress the massive release of pro-inflammatory cytokines, stabilize endothelial integrity, and thereby attenuate heat-induced BBB breakdown [8,14]. In our patient, this neurovascular compromise clinically manifested as depressed mentation upon presentation, rapidly progressing to recurrent generalized tonic–clonic seizures, partial seizures, and nystagmus—hallmark signs of severe thermal encephalopathy [9]. Given this refractory neurological deterioration, the prompt initiation of these targeted neuroprotective therapies was critical to prevent irreversible central nervous system damage and impending brain herniation.

Consequently, this case suggests that the prophylactic use of broad-spectrum antibiotics should be considered in severe heatstroke patients at risk for bacterial translocation. Moreover, when clinical signs indicate severe brain injury or refractory seizures, targeted therapies including dexamethasone, 7.5% hypertonic saline, and mannitol can serve as viable therapeutic options to manage intracranial pressure and mitigate neuroinflammation.

While the concept of a strict “golden window” is well-established in human medicine [11,12], and large-scale canine epidemiological studies similarly emphasize that early cooling is the primary determinant of survival [3,6,13], applying these temporal frameworks directly to feline heatstroke requires caution. Although the successful outcome in the present case highlights the clinical plausibility that rapid initiation of cooling and targeted intensive care are highly beneficial, definitive species-specific prognostic factors cannot be established from a single case report. Ultimately, the detailed rationale behind the therapeutic choices described herein aims to provide a useful clinical context for managing similar, rarely encountered feline emergencies.

The primary limitation of this report is the absence of precise data concerning the environmental exposure conditions, including the specific clothes dryer settings, maximum internal temperature, and exact duration of entrapment prior to discovery. Furthermore, the inherent nature of a single case report precludes the application of robust statistical analyses to establish definitive prognostic factors. Despite these constraints, reports of feline non-exertional heatstroke remain exceedingly rare in the veterinary literature. Consequently, future large-scale, multi-center studies are warranted to identify reliable prognostic indicators and establish evidence-based, species-specific treatment guidelines. Finally, evaluating the long-term impacts in heatstroke survivors is critical for improving post-recovery monitoring protocols.

## 4. Conclusions

In conclusion, this case demonstrates that severe non-exertional heatstroke with concurrent mechanical trauma can occur in felines due to household clothes dryer entrapment. Despite the profound pathophysiological cascade initiated by extreme thermal stress, immediate recognition, rapid active cooling, and aggressive hemodynamic and neuroprotective interventions can successfully halt the progression to MODS. Furthermore, the sustained elevation of acute-phase proteins, particularly fSAA, highlights the systemic inflammatory nature of this condition and the utility of serial biomarker monitoring. Beyond intensive medical management, the present case underscores the need for targeted owner education regarding hidden household hazards, such as clothes dryers. Further multi-center studies are warranted to establish definitive prognostic biomarkers and to optimize evidence-based resuscitation guidelines specifically in feline medicine.

## Figures and Tables

**Table 1:** Serial laboratory findings of the feline patient during hospitalization.

Parameter	Reference Interval	Day 1	Day 2	Day 3	Day 4	Day 5	Day 11
HCT (%) *	30.3–52.3	63.1	33.7	27.2	27.7	27.0	32.8
WBC (×10^3^/μL)	2.87–17.02	12.42	33.31	26.04	22.70	18.88	11.66
PLT (×10^3^/μL)	151–600	194	146	117	121	127	491
ALT (U/L)	12–130	3188	1778	1136	840	406	22
AST (U/L)	0–48	4018	1997	1887	1630	528	40
ALP (U/L)	14–111	55	13	14	16	13	-
GGT (U/L)	0–4	0	0	-	0	0	-
Total bilirubin (mg/dL)	0.0–0.9	0.8	0.4	1.1	0.3	0.2	
BUN (mg/dL)	16–36	20	17	15	13	-	-
CREA (mg/dL)	0.8–2.4	1.4	0.6	0.8	0.9	-	-
fPL	0–3.5	-	-	-	-	-	-
fSAA (μg/mL)	0–5	-	5.3	16.1	18.8	17.3	<5.00
CK (U/L)	0–314	>20,000	>20,000	>20,000	8614	4154	-
PT (s)	13–20	19.1	-	-	15.6	-	-
aPTT (s)	96–124	111.5	-	-	101	-	-

* Abbreviation: ALP, alkaline phosphatase; ALT, alanine aminotransferase; AST, aspartate aminotransferase; GGT, gamma-glutamyl transferase; fSAA, feline serum amyloid A; PT, prothrombin time; aPTT, activated partial thromboplastin time; HCT, hematocrit; PLT, platelet count; WBC, white blood cell count; CK, creatine kinase; fPL, feline pancreatic lipase; BUN, blood urea nitrogen; CREA, creatinine. -, not measured.

## Data Availability

The original contributions presented in this case are included in the article. Further inquiries can be directed to the corresponding author.

## Abbreviations

The following abbreviations are used in this manuscript:

fSAA	Feline Serum Amyloid A
SIRS	Systemic Inflammatory Response Syndrome
MODS	Multiorgan Dysfunction Syndrome
SBP	Systolic Blood Pressure
CRI	Continuous Rate Infusion
HCT	Hematocrit
ALT	Alanine Aminotransferase
AST	Aspartate Aminotransferase
FiO2	Fraction of Inspired Oxygen
CK	Creatine Kinase
BBB	Blood–Brain Barrier
DIC	Disseminated Intravascular Coagulation
AKI	Acute Kidney Injury
